# An Overview of Autonomous Vehicles Sensors and Their Vulnerability to Weather Conditions

**DOI:** 10.3390/s21165397

**Published:** 2021-08-10

**Authors:** Jorge Vargas, Suleiman Alsweiss, Onur Toker, Rahul Razdan, Joshua Santos

**Affiliations:** 1 Department of Engineering Technology, Middle Tennessee State University, Murfreesboro, TN 37132, USA; 2 Global Science & Technology (GST) Inc., Greenbelt, MD 20770, USA; 3 Advanced Mobility Institute (AMI), Florida Polytechnic University, 4700 Research Way, Lakeland, FL 33805, USA; otoker@floridapoly.edu (O.T.); rrazdan@floridapoly.edu (R.R.); jsantos6393@floridapoly.edu (J.S.)

**Keywords:** autonomous vehicles, sensors, perception, weather, camera, LiDAR, RADAR, GNSS

## Abstract

Autonomous vehicles (AVs) rely on various types of sensor technologies to perceive the environment and to make logical decisions based on the gathered information similar to humans. Under ideal operating conditions, the perception systems (sensors onboard AVs) provide enough information to enable autonomous transportation and mobility. In practice, there are still several challenges that can impede the AV sensors’ operability and, in turn, degrade their performance under more realistic conditions that actually occur in the physical world. This paper specifically addresses the effects of different weather conditions (precipitation, fog, lightning, etc.) on the perception systems of AVs. In this work, the most common types of AV sensors and communication modules are included, namely: RADAR, LiDAR, ultrasonic, camera, and global navigation satellite system (GNSS). A comprehensive overview of their physical fundamentals, electromagnetic spectrum, and principle of operation is used to quantify the effects of various weather conditions on the performance of the selected AV sensors. This quantification will lead to several advantages in the simulation world by creating more realistic scenarios and by properly fusing responses from AV sensors in any object identification model used in AVs in the physical world. Moreover, it will assist in selecting the appropriate fading or attenuation models to be used in any X-in-the-loop (XIL, e.g., hardware-in-the-loop, software-in-the-loop, etc.) type of experiments to test and validate the manner AVs perceive the surrounding environment under certain conditions.

## 1. Introduction

**Figure 1:**
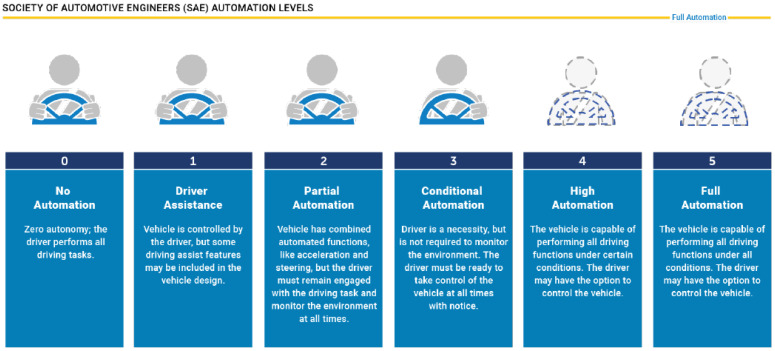
Society of automotive engineers automation levels.

**Figure 2:**
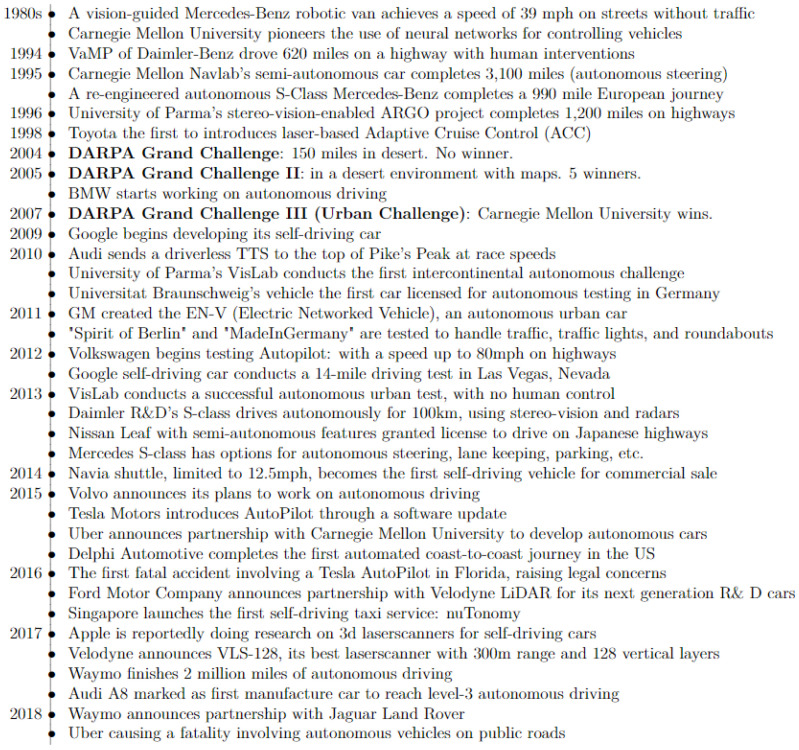
A brief summary of influential events in the history of autonomous driving.

According to the Society of Automotive Engineers (SAE), the Advanced Driver-Assistance System (ADAS) is a six-tiered system that categorizes the different levels of autonomy. It ranges from vehicles being solely human-driven to those that are completely autonomous or self-driving, as shown in Figure 1 [1]. In order to achieve higher autonomy levels, autonomous vehicles (AVs) must rely on a combination of sensors and software to perceive the surrounding environment and navigate without any human intervention. Currently, cutting-edge sensor technologies are rapidly growing to improve autonomous transportation and mobility that is safe for pedestrians and riders [2]. This has motivated more researchers and engineers, from a variety of fields and backgrounds, to engage in this process and to address all the interconnected challenges that accompanies it. Figure 2 provides a brief summary of the influential events in the history of AVs [3,4].

As a result of the growing interest in AVs, several testing and validation procedures have been developed to optimize their performance for maximum safety before being deployed onto public roadways and infrastructures [5]. AVs can be tested in simulations or in the real world. Engineers can employ actual vehicles instead of models in physical tests, which provide realistic testing settings. However, due to the high risk associated with such tests, regulations restrict their use in densely populated areas, such as cities. Furthermore, the probability of encountering edge cases is low, and even when such cases occur, the repeatability of these cases represents a challenge. According to recent reports [6,7], conducting empirical field experiments to validate the safety of AVs in an acceptable timescale is unfeasible. As a result, verifications and testing in a virtual environment have the ability to bridge the gap and enable AV systems to be evaluated in a rigorous, controlled, and rapid manner. Nevertheless, the research community recognizes the challenge of generating realistic scenarios that mimics the physical world while depending solely on software [8].

For instance, according to the National Highway Traffic Safety Administration (NHTSA), over 5,891,000 vehicle crashes occur each year on average, with approximately 1,235,000 being attributed to adverse weather conditionsm such as snow, rain, fog, and strong winds. Approximately half (~46%) of weather-related accidents are caused by rain, and approximately ~17% are caused by snow. Therefore, considering the effects of different weather conditions on the response of AV sensors can significantly improve the validity of simulated tests and generate more realistic scenarios. This paper presents a comprehensive literature review of the effects of different weather phenomena on an AV’s capability to perceive their surroundings. The details of the technological aspects involved in the development of AVs are discussed, specifying the different principle of operation for AV sensors ecosystem. In addition, the electromagnetic properties of these sensors are mapped to various weather conditions in a quantitative fashion. The remainder of this paper is organized as follows: Section 2 details the technologies normally used in AV sensors to capture information about the surrounding environment; Section 3 discusses the weather effects on AV sensors functionality; Section 4 describes the synopsis of automotive sensor strengths; and Section 5 includes our conclusions and future work.

## 2. Autonomous Vehicles Sensors Ecosystem

**Figure 3:**
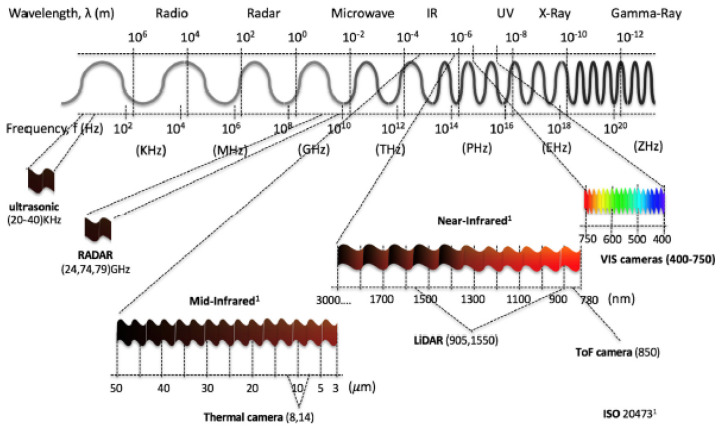
Electromagnetic spectra used by sensors on autonomous vehicles.

**Figure 4:**
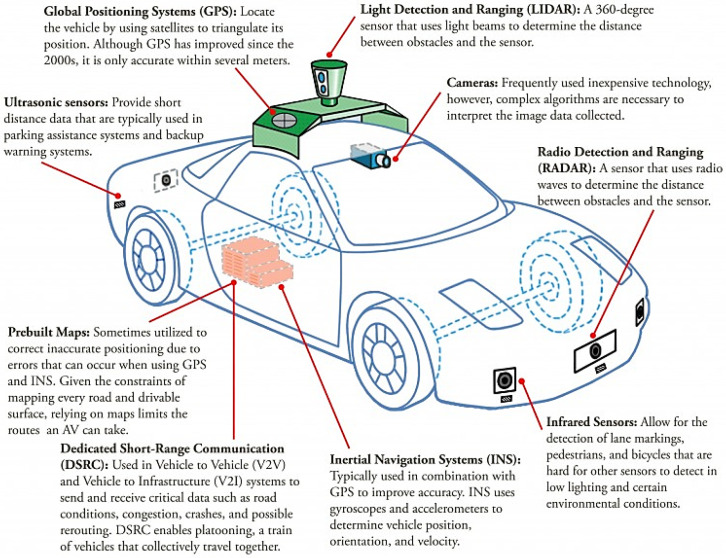
Autonomous vehicles sensor ecosystem (Image source: The Economist).

This section presents the most representative sensors that make up AVs sensors ecosystem: RADAR, LiDAR, ultrasonic, global navigation satellite system (GNSS), and cameras. These sensors measure wave sources and detect various physical phenomena. They have distinct properties that enable them to perform different tasks under specified conditions. In this work, we focused on the part of the electromagnetic spectrum that they use in their operation. This will shed the light on their vulnerabilities to degraded environments, such as adverse weather conditions. Figure 3 depicts the electromagnetic spectrum and the spectral ranges used by AV sensors investigated in this work. Furthermore, Figure 4 shows a top-level description of the AV sensors suite with a brief description.

### 2.1. RADAR

RADAR is an acronym for Radio Detection and Ranging technology, which is a device that uses radio waves for object-detection within a certain range. When the transmitted waves intercept with an object along its propagation path, they are reflected by its surface where the RADAR antenna collects the backscattered signal (echo) within its field of view (FOV). The round-trip delay time, together with the known velocity of radio waves, provides a precise determination of the object’s distance and velocity from the RADAR system. The RADAR range equation that relates the received echo power (*P_r_*) to the distance of an object R meters away is shown in Equation (1) [9]:

(1) $$P_{r}\left(R\right)=\frac{P_{t}G^{2}\lambda^{2}\sigma L}{{\left(4\pi \right)}^{3}R^{4}}$$

where *P_t_* is the transmitted power, *G* is the gain, λ is the wavelength, σ is the cross section of the target, and L represents all the losses lumped together, including multipath, atmospheric, and environmental losses.

**Figure 5:**
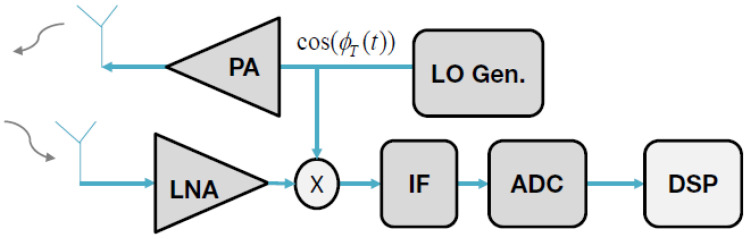
High-level block diagram of an FMCW RADAR system.

The RADAR systems for AVs operate at 24, 74, 77, and 79 GHz (millimeter wave (MMW)), which are separated out to work for short-range, medium-range, and long-range RADAR (SRR, MRR, and LRR, respectively) [9]. An LRR can be implemented to detect far targets or objects in front of the ego car, and MRR and SRR, on the other hand, are used for parking assistance or side view detection [10]. Among a plethora of RADAR technologies available nowadays, linear Frequency-modulated continuous-wave (L-FMCW) RADARs are commonly used in AVs due to their simplicity. Figure 5 shows a top-level block diagram of an FMCW RADAR, where the voltage-controlled oscillator (VCO) module generates an L-FMCW chirp signal that is amplified by the power amplifier (PA) and transmitted by the antenna. The receiving antenna captures the echo signal and the low noise amplifier (LNA) amplifies it before mixing it with the VCO signal in order to generate the intermediate frequency (IF) or beat signal. The analog to digital converter (ADC) then digitizes the signal and passes it to the digital signal processing (DSP) module.

**Figure 6:**
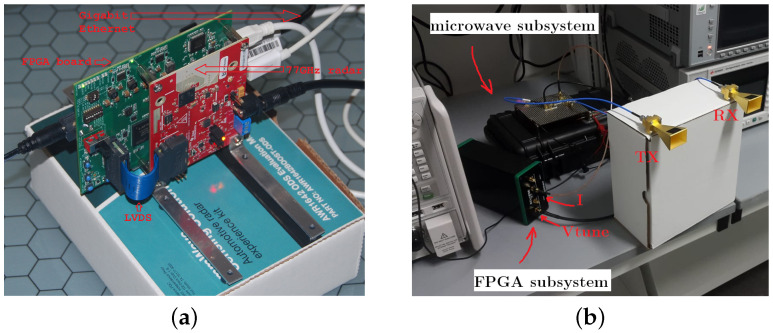
(**a**) A 77 GHz radar with PCB antennas and (**b**) 24 GHz radar with horn antennas.

Currently, most AV RADAR systems use an array of micro antennas capable of generating a set of antenna lobes. For instance, a 77 GHz radar with printed circuit board (PCB) antennas and a 24 GHz radar with horn antennas are shown in Figure 6 [11]. This has become more common with system-on-chip (SOC) architectures, which allows digital beam forming, among other techniques, to restrict the receiver’s spatial FOV and reduces the electromagnetic interference from other active sensors operating at similar center frequencies. The advancements of smart vehicles have triggered the use of this type of device in the automotive sector in order to improve safety. Adaptive cruise control (ACC), collision avoidance systems (CAS), blind spot detection (BSD), and lane change assist (LCA) are some examples. Matlab was used to create the initial step in establishing a complete testbed for AV sensor testing and verification, with an emphasis on radar systems under all environmental conditions of relevance [12].

### 2.2. LIDAR

**Figure 7:**
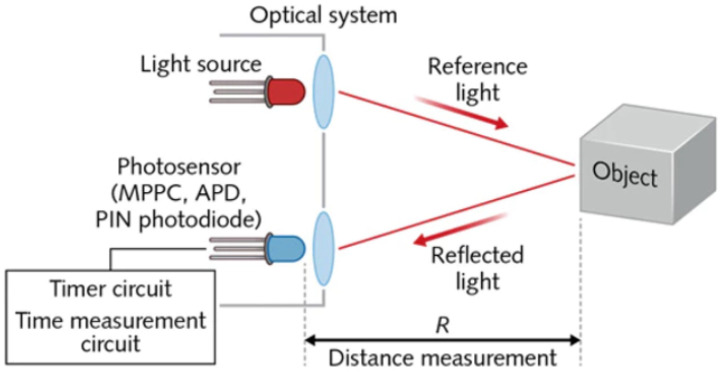
High-level block diagram for time-of-flight LiDAR system (Image source: LaserFocusWorld).

LiDAR is an acronym for Light Detection and Ranging, which is a technology developed in the 1970s to be deployed on space platforms and airborne platforms. In a similar fashion to RADARs, LiDAR systems base their operation on measuring the time it takes a pulse of light, in infrared or near-infrared ranges, emitted from a laser diode until it is received by the system’s receiver, which is also known as the time-of-flight (ToF) principle. In ToF technology, the LiDAR generates a pulse of light with a specified duration (τ) that, at the time of emission, triggers the internal clock in a timing circuit. The reflected light pulse from the target is detected by a photodetector, which produces an electrical output that disables the clock. The distance to the reflection point may be calculated by using this electronically measured round-trip ToF (Δ*t*) [13], as indicated in Equations (2) and (3):

(2) $$P\left(R\right)=P_{0}\rho \frac{A_{0}}{\pi R^{2}}\mu_{0}\exp-2\gamma R$$

(3) $$R=\frac{1}{2n}c\Delta t$$

where *P*_0_ is the optical peak power of the emitted laser pulse, ρ is the reflectivity of the target, *A*_0_ is the receiver’s aperture area, $\mu_{0}$ is the detection optics spectral transmission, γ is the atmospheric extinction coefficient, *c* is the speed of light in a vacuum, and *n* is the index of refraction of the propagation medium (~1 for air). Figure 7 demonstrates a high-level block diagram for ToF LiDAR.

The main wavelengths used in LiDARs are 905 nm and 1550 nm, which are determined by atmospheric transmission windows and the availability of high-power pulsed sources [13]. At the early development stages of AVs, 905 nm pulsed LiDAR systems were chosen for the task due to their availability. However, these systems have some serious limitations, such as high cost, inefficient mechanical scanning, interference from other light sources, and eye-safety power restrictions that limit their detection range to ~100 m. This prompted the shift to the retinal-safe 1550 nm band, because the water in the atmosphere begins to absorb energy from 1400 nm, which allows pulse powers high enough to range from 200 to 300 m [13]. LiDARs used for vehicles belong to Class-1 [14] and are safe under all conditions of normal use.

Creating a three-dimensional (3D) profile (typically 360° in azimuth × 20° in elevation) of the environment surrounding the AV requires a raster-scanned laser beams (scanning LiDAR) or flooding the scene with light and collecting the returns (flash LiDAR). For scanning LiDARs, the platform emits pulses from a set of diodes mounted on a rotating pod or by using a rotating multi-faceted mirror. The moving parts in these designs (rotates at 300–900 rpm) represent points of high failure rate in rough driving environments. Other approaches that can reduce the need for mechanical steering include the use of microelectromechanical systems (MEMS) mirror to steer the lens electrically or the use of optical phased array (OPA) technology. On the other hand, flash LiDARs flood the scene within the FOV of the detector with light. The detector is an array of avalanche photodiodes (APDs) where each independently measures ToF to the target feature imaged on that APD.

### 2.3. Ultrasonic Sensors

Ultrasonic sensors are suitable for many detection tasks in industrial applications. They have the capability to detect objects that are solid, liquid, granular, or in powder form. Ultrasonic sensors rely on sonic transducers to transmit sonic waves in the range of 40 kHz to 70 kHz for automotive applications. This frequency range is beyond the audible range for humans, which makes it safe for human ears. This is an important factor given that a cars’ parking system can generate more than 100 dB of sound pressure to assure clear reception, which is equivalent to the audible sound pressure from a jet engine.

Most ultrasonic sensors are based on the principle of measuring the ToF of sonic waves between transmission and reception. This measured ToF is then used to calculate the distance (*d*) to an object or a reflector within the measuring range, as shown in Equation (4).

(4) $$d=Speed\;of\;Sonic\;Wave\times \frac{ToF}{2}$$

**Figure 8:**
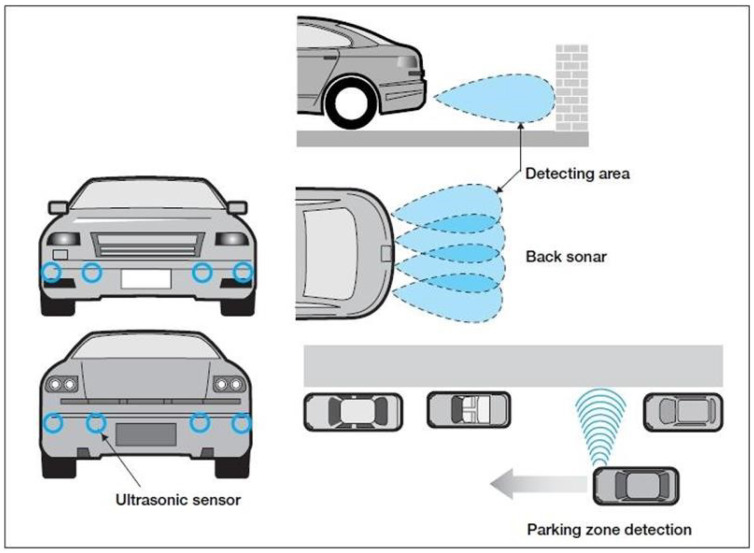
Applications of ultrasonic sensors in vehicles (Image source: newelectronics).

Sonic waves travel in air at ~340 m/s and are a function of air temperature, pressure, and humidity (for each degree Celsius, the speed of sound increases by 0.6 m/s). The time it takes a sonic wave to travel 1 m is approximately 3 × 10^−3^ s, as opposed to 3.3 × 10^−9^ s for light and radio waves. These several orders of magnitude differences allow the use of low speed signal processing in ultrasonic systems. However, pressure based atmospheric conditions can debilitate the overall performance of ultrasonic sensors [15,16], which promotes the use of SRRs and other technologies instead. Figure 8 demonstrates the application of ultrasonic sensors in vehicles.

### 2.4. GNSS

GNSS is the most widely used technology for providing accurate position information on the surface of the earth. The best-known GNSS system is the Global Positioning System (GPS), which is a U.S. owned utility that provides users with positioning, navigation, and timing (PNT) services. The free, open, and dependable nature of GPS made it an essential element of the global information infrastructure that affects every aspect of modern life.

The GPS was developed by the U.S. Department of Defense (DoD) in early 1970 and is divided into three segments: the space segment, the control segment, and the user segment. The GPS system’s space and control elements are developed, maintained, and operated by the US Air Force [17]. The space segment consists of 31 operational satellites of which at least 24 are available 95% of the time. These satellites fly in medium earth orbit (MEO) at an altitude of 20,200 km, and each satellite orbits the earth twice daily. Its configuration allows any receiver located on the earth’s surface to receive signals in the L-band and some of the S-band frequency range, from 6–12 satellites. The control segment corresponds to a global network of ground facilities that track GPS satellites, analyze their broadcasts, perform analysis, and provide orders and data to the constellation; and the user segment refers to the process of dividing into distinct groups based on shared characteristics.

The operating principal of the GNSS is based on the ability of the receiver to locate at least four satellites, to calculate the distance to every single one of them, and then uses this information to identify its own location by using a process called trilateration. It is worth mentioning that GNSS signals suffer from several errors that degrade the accuracy of the system, such as the following: (1) timing errors due to differences between the satellite atomic clock and the receiver quartz clock, (2) signal delays due to propagating through the ionosphere and troposphere, (3) multipath effect, and (4) satellite orbit uncertainties. In order to improve the accuracy of current positioning systems on vehicles, data from satellites are merged with data from other vehicle sensors (e.g., inertial measurement unit (IMU), LiDARs, RADARs, and cameras) to achieve reliable position information.

### 2.5. Camera

Self-driving vehicles may rely heavily on cameras to perceive the surrounding environment. According to the electromagnetic spectrum, most cameras can be classified as visible (VIS) or infrared (IR). VIS cameras (e.g., monocular vision [18,19,20,21] and stereo vision [22,23]) capture wavelengths that ranges from 400 to 780 nm, similarly to human eyes. They are mostly used due to their low cost, high resolution, and their capability to differentiate between colors. Combining two VIS cameras with a predetermined focal distance allows stereo vision to be performed; hence, a 3D representation of the scene around the vehicle is possible. However, even in a stereoscopic vision camera system, the estimated depth accuracies are lower than the ones obtained from active range finders such as RADARs and LiDARs.

IR cameras work with infrared wavelengths ranging between 780 nm and 1 mm. They can be extended to the near-infrared (NIR: 780 nm–3 mm) and the mid-infrared (MIR: 3–50 mm; known as thermal cameras) [24,25] for certain applications. IR cameras are less susceptible to weather or lighting conditions, and it can overcome some of the VIS cameras shortcomings in situations where there are peaks of illumination (e.g., at the exit of a tunnel). In addition, they can be used for warm body detection, such as pedestrians and animals [26,27,28,29].

Moreover, NIR cameras can be used for range detection by using the ToF principle and the phase difference between the transmitted and the received light pulses. Depending on the number of light emitting diodes (LEDs) utilized in the LED array, the distance ranges from 10 m for interior scenes to roughly 4 m for outdoor scenes.

### 2.6. AV Sensors Performance Comparison

As briefly described in this section, different sensors have different advantages and disadvantages that play a role in their deployment on AVs and in fusing their responses in the object identification model needed in self-driving cars. Table 1 summarizes some of the main characteristics of different AV sensors. Their vulnerability to weather effects remains the focus of this paper and will be discussed in the following section.

## 3. Weather Effects

Under ideal operating conditions, AV sensor technology should perform as expected when it comes to perceiving the surrounding environment and executing necessary actions. However, adverse weather conditions (e.g., rain, snow, fog, unfavorable lighting conditions, etc.) can impose serious challenges to AV sensors and the algorithms distilling information from them. This section will summarize the effects of several common adverse weather phenomena on AV sensors in a quantitative manner.

### 3.1. Precipitation (Rain, Snow, Hail, Sleet)

Precipitation is water (liquid or frozen) that falls back to the ground after condensing in the colder atmosphere. A droplet’s size and distribution defines the intensity of precipitation (measured in millimeters/hour (mm/hr)), which in turn affects the mechanism’s electromagnetic signals that propagate through the precipitated medium. According to [30], the maximum diameter a rain droplet can have is 6 mm. If the rain droplet diameter exceeds this amount, the air resistance coupled with the terminal velocity of the droplet will exceed its cohesive force and tear it into smaller pieces.

According to Mie’s solution to Maxwell’s equation, any transmission wavelength (λ) that is similar or smaller to the droplet diameter of 6 mm will be subject to Mie scattering [31]. Mie scattering can affect the propagation of the EM signal in two ways: first, the absorption of EM energy by water drops and vapor causing attenuation; and second, the rain volume back scattering or rain clutter can generate false alarms or mask actual targets in front of the sensor.

**Figure 9:**
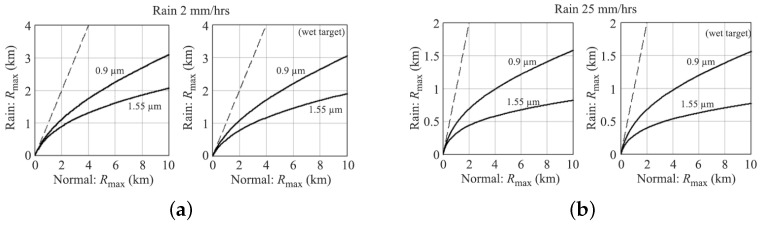
The visibility effects of differing rain intensities and target wetness on 905 nm and 1550 nm waves vs. normal conditions for (**a**) 2 mm/h and (**b**) 25 mm/h rain rate.

**Figure 10:**
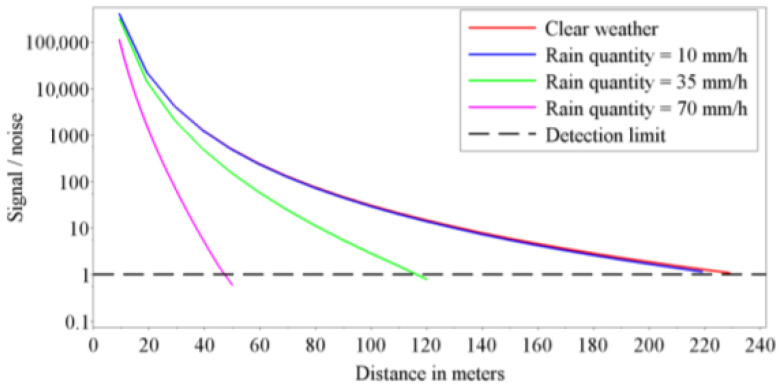
Variation of the signal/noise ratio as a function of the rain rate and distance between LiDAR and target for a rain droplet radius equal to 3 mm.

LiDARs transmitting in the 905 nm and 1550 nm wavebands will be heavily affected by Mie scattering from rain. Figure 9 shows the visibility deterioration on LiDAR during different rain intensities [13]. As can be observed from Figure 9a, LiDAR visibility in clear conditions, which should have been ~2 km at these wavebands, has been deteriorated to approximately 1.2 km for the 905 nm band and 0.9 km for the 1550 nm band in 2 mm/h rain. When the rain rate increases to 25 mm/h, the 2 km visibility drops to 0.7 km and 0.45 km for wavelengths 905 nm and 1550 nm, respectively, as illustrated in Figure 9b. The wetness of the target decreases the visibility by an additional addend of 0.1 km. However, within the range of 250 m usually required for rangefinders on AVs, LiDAR susceptibility to rain is not as noticeable until more severe rain rates occur, as shown in Figure 10 [30].

**Figure 11:**
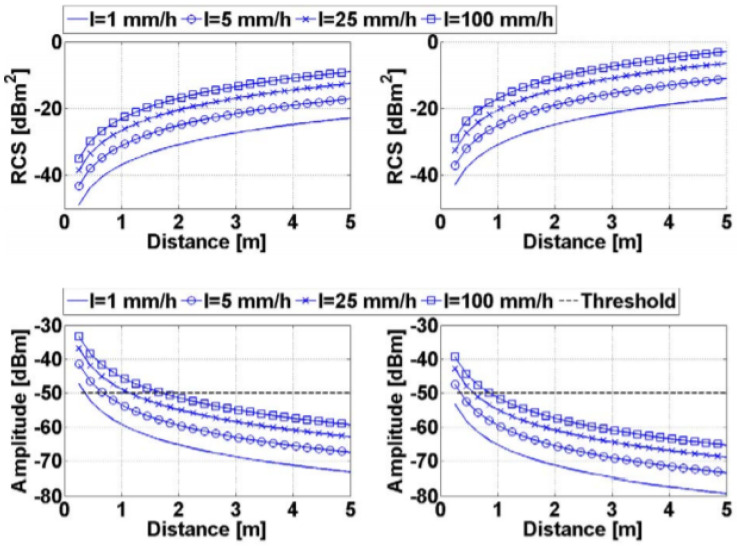
Rain RCS (**top**) and received power (**bottom**) for narrow beam (**left**) and wide beam (**right**) for different rain rates.

For 77 GHz RADAR systems used in AVs (λ ≈ 3.9 mm), the effect of attenuation is not significant at short distances [32]. It ranges from 0.0016 dB/m for 1 mm/h to 0.032 dB/m at 100 mm/h [33]. However, rain backscattering or rain clutter can decrease the maximum range of detectability, as discussed in [34,35]. Moreover, since the received backscatter from the rain depends on *R*^2^ instead of *R*^4^ for the target echo where *R* is the range, rain clutter can exceed the detection threshold and results in false alarms. Figure 11 summarizes the results of an experiment conducted by [33] in order to evaluate the effects of rain volume backscattering on 77 GHz RADARS used in AVs. It demonstrates rain RADAR cross section (RCS) and its received power for different rain rates and ranges and for narrow and wide beam RADAR systems.

GNSS is generally not affected by local weather conditions. These satellite systems operate at a frequency (approximately 1.575 GHz) that is mostly unaffected by weather [36]. However, the windshield wipers will occasionally block reception, making it impossible for a GNSS device to identify a complete navigation data string from satellites. As a result, the GNSS receiver may not properly decode the incoming string and may provide erroneous data [37].

Camera systems onboard AVs relies on scene brightness to determine the intensity of image pixels. Different weather conditions can introduce sharp intensity fluctuations that can result in quality degradation of images and videos. For instance, snow and heavy rain can obscure edges of an object, rendering it unrecognizable. Fortunately, some digital image processing techniques can mitigate the effect of precipitation and improve images quality under dynamic weather conditions [38].

### 3.2. Fog

Fog is a visible aerosol consisting of small water droplets suspended in the air or near Earth’s surface [39]. It is considered as a low-lying cloud. This weather phenomenon typically begins around midnight and mostly dissipates after sunrise once atmospheric temperatures raises and relative humidity decreases.

Dust or air pollution must be present in the air for fog to occur. Water vapor condenses around these small solid particles, generating droplets ranging in size from 1 to 20 microns [40]. This being the case, LiDAR systems, for which its operating wavelengths are less than fog particles, will be subject to Mie scattering. In addition to the adverse effects from scattering, water absorption has a huge impact on the NIR spectral band. Water’s contribution to the extinction coefficient for the 905 nm and the 1550 nm are 0.075 cm^−1^ and 10.8 cm^−1^, respectively. According to M. Hadj-Bachir et al. and J. Wojtanowski et al., under normal conditions, the overall extinction coefficient between these two frequencies are double one to another juxtaposed to the two magnitudes of difference observed from fog’s presence.

Ultrasonic sensors will not be directly affected by scattering compared to most of other sensors. However, being a system working with sound waves, air constitution and temperature would influence its performance. Nonetheless, given the fact that ultrasonic sensors are used for applications that required very short-range detection, the effect of precipitation is very minimal.

**Figure 12:**
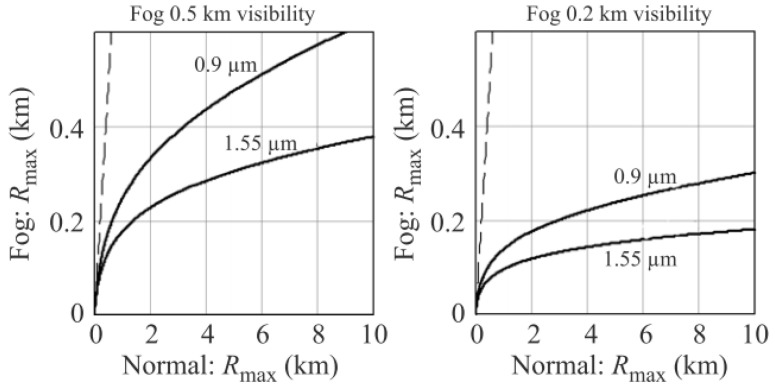
Range degradation curve observed when 905 nm and 1550 nm are subjected to 0.5 km and 0.2 km visibility fog.

The range degradation curves presented by J. Wojtanowski et al. in Figure 12 show the severity of the signal attenuation seen in different ratings of fog. What should be 0.5 km of visibility has diminished to about 0.20 km and 0.12 km for the 905 nm and 1550 nm wavelengths, although they have the same maximum range performance under normal conditions. Moderate continental fog and heavy maritime fog can attenuate NIR signals up to 130 dB/km and 480 dB/km, respectively [36]. Fog would exhibit slight Rayleigh scattering on millimeter waves due to the extreme magnitude difference of particle to wave size. Fog might indirectly affect RADAR should temperature requirements be met by condensing on the radome or target in question and, thus, emulating what was explained in the above section. The camera, on the other hand, is heavily affected by Mie scattering since its operating wavelengths (400–750 nm) are much smaller than fog particle size. Reference [37] examines a camera’s ability within a fog chamber of 2 micron and 6 micron size fog particles. It was noted that visibility was reduced to 30 m compared to its ideal 60 m. The presence of air-light should also be taken into consideration since fog facilitates it. Air-light can be defined as the scattering of light from the interference of particles. With air-light interference, objects within the immediate vicinity of the light source are impossible to perceive. As the proximity increases, the perception of other objects is left to how the air-light interferes with the camera. Object distinction becomes increasingly difficult within fog and becomes more reliant on the reflectivity of the objects in question. The higher the reflectivity of an object, the darker it would appear in fog [37]. Ultrasonic sensors, relying on air constitution, would also be affected by fog. Depending on the type of fog, the air’s water density can range from 0.01 to 0.2 g/cm^3^. This interaction will be discussed further in the humidity section.

### 3.3. Humidity

Humidity is broken down into two different types: absolute and relative. Absolute humidity is the amount of water vapor in a cubic meter of air. Depending on the temperature of the environment, the amount of water vapor and miscellaneous particles that can be contained within a volume could vary. When observing absolute humidity with the current temperature, relative humidity is attained. Relative humidity is 100 percent or saturated when the dew point temperature and actual temperature match. With this in mind, the ratio of the actual vapor pressure to the saturated vapor pressure will also be the relative humidity [41]. If saturation is achieved along with certain weather conditions, fog formations could be facilitated. Since pressure is directly affected by humidity, humidity should affect ultrasonic sensors. The levels of humidity have a variable effect on the attenuation of ultrasonic waves. This effect is not necessarily the same for all frequency to humidity levels. For example, at 200 kHz, maximum attenuation is observed at saturation but at 60 kHz, max attenuation is observed at 60% humidity. The latter frequency is relatively close to modern AV sensor frequencies. In conditions of no humidity, attenuation is approximately 0.25 dB/ft. Another phenomenon observed by [39] was at 20 °C; attenuation seemed to rise sharply.

Based on water’s high absorption coefficient, high humidity should have a noticeable contribution to the attenuation of LiDAR performance. Under normal conditions, the extinction coefficient of the 905 nm is typically about double the 1550 nm’s one. Table 2 shows the atmospheric extinction coefficients subjected to varying levels of humidity, as reported by J. Wojtanowski et al. As can be observed, the extinction coefficients of the 905 nm and the 1550 nm approximately keep their ratios the same. That being said, different levels of humidity have negligible effects on the performance of LiDAR. Since LiDAR is not affected by water vapor content of humidity, RADAR, being a more robust system, will also not be affected by humidity levels [33].

### 3.4. Lightning

It is important for the AVs to operate with electromagnetic stability on its own in ideal conditions, since it will be making use of technology that requires electricity and electromagnetism [40]. Other unpredictable conditions outside of electrical interference need to be considered; one of the biggest concerns of this type is lightning.

Lightning is the weather phenomenon where large electrical discharge occurs and balances itself again by producing a momentary flash of up to 30,000 K in under 10 microseconds [42]. Since clouds are not specifically monitored to observe when lightning might occur and where it may travel, all AV sensors are in danger, especially if located on the exterior of the vehicle. Depending on the car material and structural composition, the surface of the car will hold the electrical charge upon strike. Contrary to popular belief, the passengers may still be negatively affected if in contact with any of the vehicle’s shell as well as components connected to the shell such as the steering wheel, gear shift, doors, or windows; passengers might even be affected by interacting with technology that has electrical capability. Table 3 compares the AV equipment under lightning influence.

**Figure 13:**
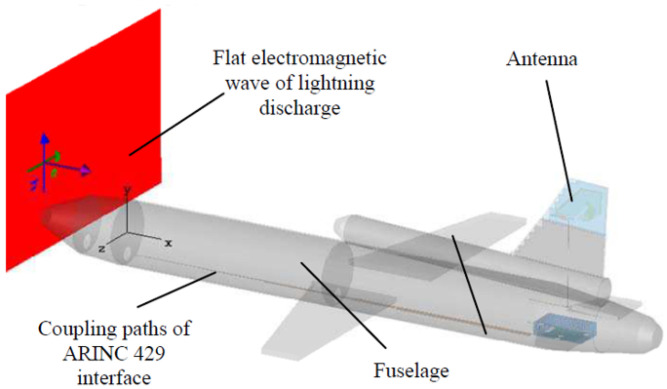
Example of a UAV model.

**Figure 14:**
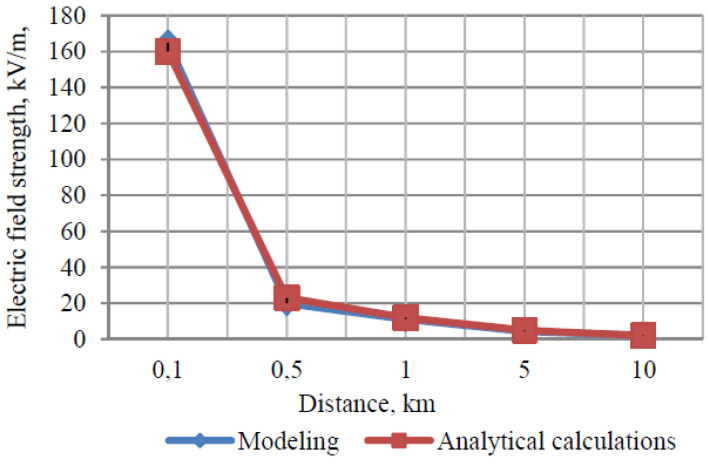
Electric field strength due to lightning.

**Figure 15:**
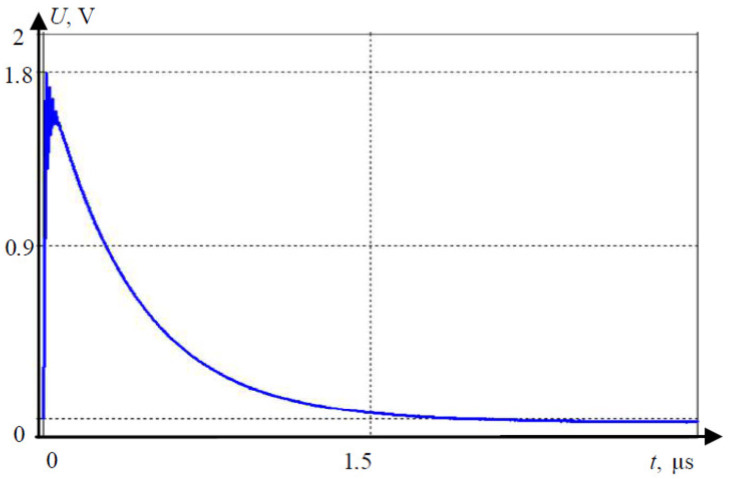
Electromagnetic disturbance example in the coupling path.

**Figure 16:**
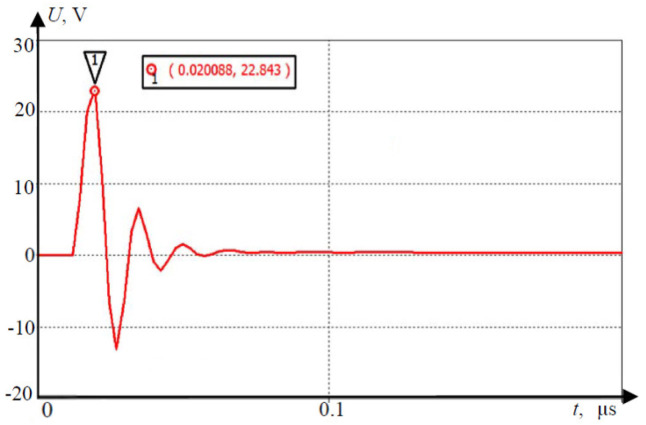
Electromagnetic disturbance example in the antenna-feeder path.

AV sensors can be affected by lightning even when not directly struck; this includes the technologies of the car, since the sensors are usually mounted to the vehicle. The electromagnetic stability can be affected by the new electromagnetic field created by the electrical discharge from the clouds. An example of unmanned aerial vehicle (UAV) produces a charged field in Figure 13; the strength of it over distance is in demonstrated in Figure 14, which decreases as distance increases [43]. Figure 15 and Figure 16 show electromagnetic disturbances in the UAV [43]. This permits us to observe how radio disturbances appear to be prevalent. Among the AV sensors, the camera is also affected visually by highly unpredictable lighting changes. The bright light can affect perception in cameras.

The lightning discharge effects can be maintained by material choice for low electrical conductivity and controllers for less noise immunity. Guaranteeing noise immunity can be performed by structural methods, circuitry methods, electromagnetic shielding, and filtering. In the end, it comes down to the electromagnetic compatibility (EMC) of the system with a natural external disturbance [40]. For the UAV example, the conclusion was that the effects of the noise immunity on radio waves are very probable, while the effects of electronic equipment are not as significant [40]. This topic could have more material available in the field or is a great place to begin simulations per device (RADAR, LiDAR, etc.).

### 3.5. Thunder

The sound aspect following lightning is thunder; they both occur when the other is present, even if the thunder is inaudible [42]. As the air has been heated along the path of electrical discharge, the excited particles produce sound in an explosion that can be heard up to 30 km away [42]. Since the speed of light is greater than the speed of sound, the noise is heard after the flash.

Even though sound can travel a long distance, the audibility can be affected by numerous factors. Humidity, wind velocity, temperature inversions, terrain features, and clouds are all noise distorters; this is important primarily for ultrasonic sensors [42]. Echoing effects can also be produced by buildings or landscapes causing multiple occurrences of the sound.

Thunder can operate at both the sonic and infrasonic ranges. The dominant frequency is 100 Hz, but infrasonic frequencies, which are below 20 Hz, are inaudible to humans [44]. Table 4 shows a list of general sound levels for a comparison to thunder. The pressure waves associated with thunder are, therefore, able to damage interior or exterior structures [44].

**Figure 17:**
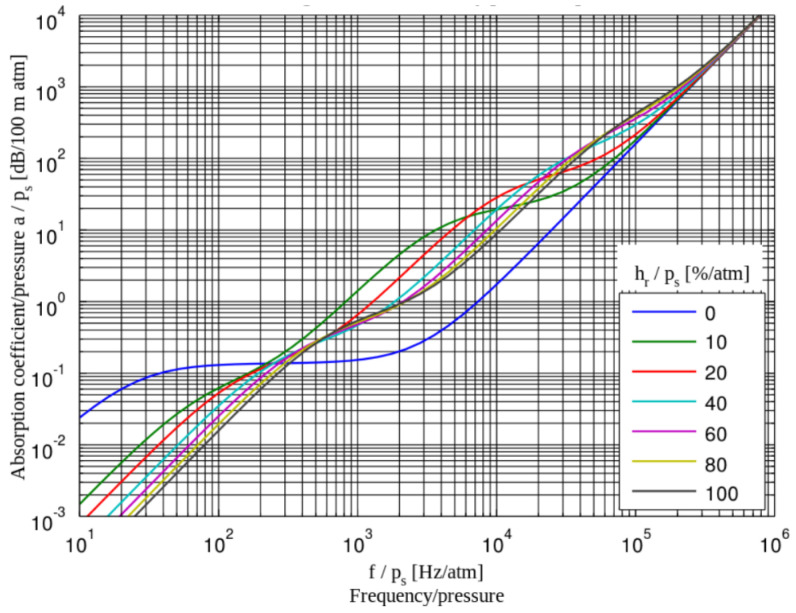
Sound attenuation dependent on relative humidity (RH) and frequency at 20 degrees Celsius.

Depending on the initial conditions such as temperature and relative humidity, the effect of thunder could be negligible with regard to sound for various sensors observed in Figure 17 [45]. However, in the case of thunder, temperature is much higher (at 30,000 K due to lightning); RADAR, LiDAR, ultrasonic, and possibly GNSS will be affected due to disturbances of wavelengths.

### 3.6. Sun Glint

**Figure 18:**
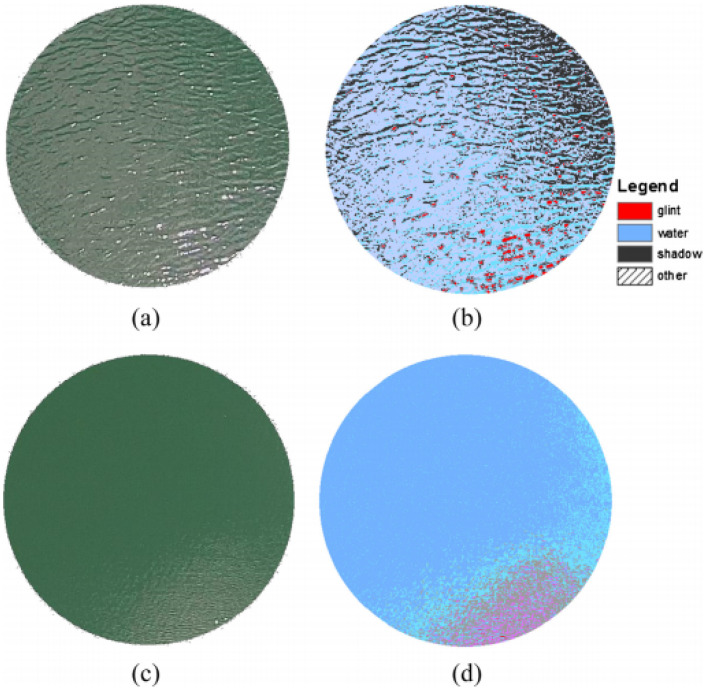
Surface sun glint patterns.

Sun glint is the surface scattering of reflectance on liquids [46]. Figure 18 shows a picture of water in (a) and (c) with a respective filtered image in (b) and (d) displaying where the glint occurs. The colors represent the following: glint in red, water in sky blue, and shadow in black [46]. This poses a problem as vision or object-detection could be faulty when receiving a light signal back at a certain size. The objects interacting with AVs and producing glint will mainly be in the precipitation. The sensors are sharply divided on the attenuation due to sun glint. There are no effects on RADAR, ultrasonic sensors, or GNSS, while LiDAR and cameras will be the most affected by this occurrence. As observed in the example above, shown in Figure 18, the larger the amount of glint, the more it will affect the readings.

### 3.7. Dust Storm

Dust storms are the formations of sand or dust walls typically caused by strong wind currents, which are typically offshoot from a large thunderstorm and/or, on some rarer occasions, the movement of pressure fronts [47]. These dust particles, for which its size can range from 2.5 to 10 microns, are pushed by the current causing visibility concerns and may also facilitate cloud growth [48]. Since visibility concerns are present, camera performance is severely debilitated relative to whether there may be poor visible conditions in the air or the camera lens becoming dirty. For the former, over the span of a year, 549 incidents involving casualties were reported to be caused by dust-related storms [47]. According to Sky Harbor International Airport, dust storms debilitate visibility to less than half a mile (0.8 km). Smoke, which shares some physical characteristics with dust storms, also cause performance issues. Energy absorption and soot particles, similarly to the dust particles, have higher effects relative to sensors with smaller wavelengths and can be around 400–750 nm in the camera’s case [49].

LiDAR is also affected negatively by smoke but not as severely as the camera [48]. Similarly to how fog or rain affects LiDAR, dust storms particles will cause Mie scattering to the 905 and 1550 nm wavelengths, since their sizes are around 2.5–10 microns. This scattering will not be as powerful, however, because water as a material has a high absorption coefficient [13]. RADAR will only be affected minorly by Raleigh scattering since particle size and wavelength differ three magnitudes.

Since ultrasonic sensors are extremely dirt-tolerant, their efficiency shines, especially in harsh working environments where process reliability is not jeopardized by dust, smoke, mist, or other impurities. However, this is only with respect to the particles contained within dust storms. Wind speeds of 6 m/s begin to disturb wave propagation [39]. That being said, more than half of the dust storms observed in Phoenix, Arizona, had maximum wind speeds between 36 and 57 mph (16–25 m/s) [47].

### 3.8. Space Weather

**Figure 19:**
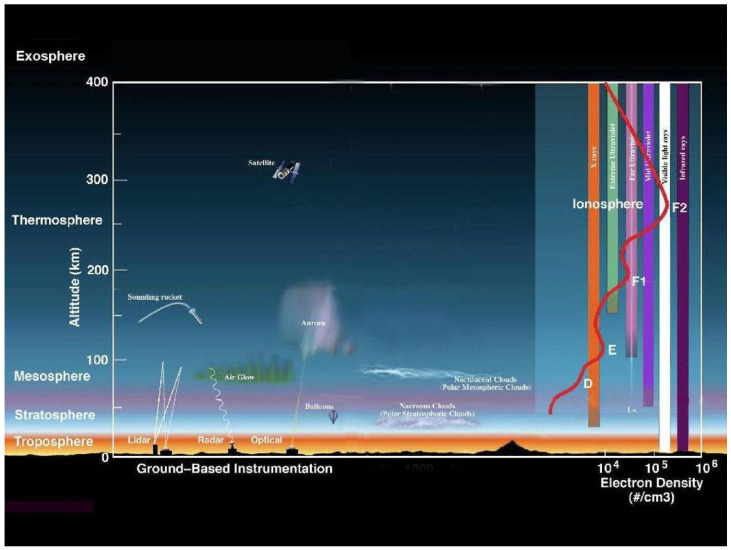
Earth’s atmospheric layers.

The earth’s atmosphere observed in Figure 19 is composed of a few layers, including the troposphere, stratosphere, mesosphere, thermosphere, ionosphere, and exosphere, with most of the planet’s weather occurring in the troposphere [50]. The previously discussed weather phenomenon mainly affects RADAR, LiDAR, ultrasonic sensors, and cameras. GNSS is an exception to the group being mainly affected by environmental conditions in our solar system (space weather) [36].

The ionosphere is abundant in electrons and ionized particles and renders radio communication possible [50]. This layer also can shift in size, depending mainly on solar influence [50]. GNSS operates in this region and can be influenced by interrupting wavelengths and objects coming in from the exosphere. The troposphere, unlike the ionosphere, has the benefit of multiple layer protection, where meteors can be disintegrated in the mesosphere and ultraviolet radiation can be absorbed and dispersed through the stratosphere’s ozone layer [50]. Technology utilized in the ionosphere is at a higher disadvantage if something were to permeate the planet’s atmosphere, observed in Table 5.

The L-Band frequencies used by GNSS are severely debilitated by space weather. Space weather, which actably accounts for the sun’s atmospheric interactions, does have an effect on range delay and loss of lock [51]. The sun has a direct effect on the ionosphere, whether that may be through ionospheric scintillations, changing the density and distribution of the total electron content (TEC), or the time of day and year. The ionosphere can be broken down into three main layers denoted as the D, E, and F regions. These regions are classified by altitude and each effect signals differently. The D-region attenuates signals inversely proportional to the frequency squared. This being the case, attenuation within the L-Band is negligible. The E and F regions cause little to no attenuation due to low air density. They tend to reflect the signal depending on the frequency and incidence angle of the transmitted wave. The Ionosphere’s index of refraction is a function of the transmitted wave frequency and the TEC of the region. At nighttime, the TEC distribution is generally lower within the E region; therefore, lower reflections and range delays occur [51]. Ionospheric scintillations have a direct effect on the E and F regions, which result in an increase in loss of lock for the GPS. Solar flares cause variations relative to the TEC, which also causes loss of lock variations [51]. Additionally, solar radio bursts negatively impact tracking performance.

## 4. Synopsis of Automotive Sensor Strengths

**Figure 20:**
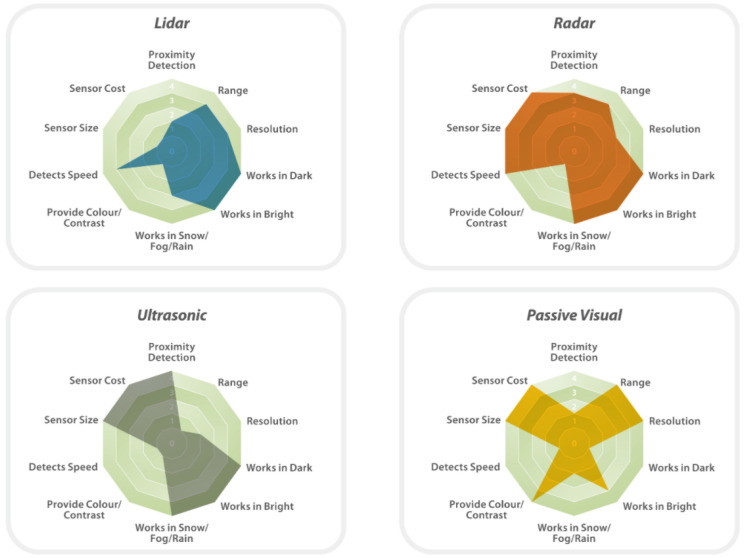
Spider diagrams showing strengths of various sensors found in automobiles. (Image source: www.cleantechnica.com; accessed on 12 March 2021).

Several strengths of different types of automotive sensors are shown in Figure 20. A variety of sensors are needed for reliability in which sensors must be placed in order to cover different ranges and speeds for high-speed driving, weather conditions, collision avoidance, and stationary safety.

LiDAR sensors are vulnerable to precipitation conditions such as snow, hail, sleet, and rain, as shown in Figure 20. However, as a short wavelength technology, LiDAR can identify small objects and provide a precise 3D monochromatic picture of an item, which RADAR may lack. The perception of objects is predicted to be severely affected by inclement weather and a decrease in detection range. As a result of the diminished contrast in intensity, there is an increase in misclassifications and even incorrect detection systems [52]. If the raindrop is very close to the laser emitter, there is a considerable possibility of erroneous detection. Since the laser beam meets a particle and causes a flash of light similar to a small surface, there will be a return of peak that is similar to that of a road item [37].

RADAR sensors can operate in varied weather conditions due to a wide spectrum wavelength and absence of mechanical moving parts, as opposed to passive visual sensors due to their lack of scope for climate range. A radio wave receiver and transmitter of the same wavelength can be used to generate radio noise, causing the device to count the speed of a moving object as zero. Then, when it comes to RADAR versus LiDAR autonomous automobile systems, both technologies are deceptive and have the same level of security [53]. Rain is the most important source of radar signal attenuation [54]. Since droplet sizes are equivalent to radar wavelengths, the rain backscatter effect was discovered to have a considerable impact on performance. The attenuation effect weakens the signal, whereas the backscatter effect adds interference at the receiver [55]. These indications are equally useful in the presence of snow or mist. When it comes to hail, millimeter-wave radar signals lose part of their power as they move through the weather [56,57]. The mathematical models for snow and mist attenuation and backscatter are the same as those for wet conditions [58].

Cameras offer reliable information about the environment and can operate in a variety of weather conditions [59]. During self-parking, a visual navigation system can identify nearby obstructions and locate the vehicle, provided that a complete and accurate full view of the surroundings is taking place in a limited space. In order to accomplish this, a combination of stereo vision techniques can be used [60]. With the use of real-time depth map extraction, a precise dense map may be built up in the sequence of depth map extraction, obstacle extraction, and fusion across multiple camera frames to achieve an accurate estimation [60].

Ultrasonic or sonar sensors are used for near-obstacle detection and as a parking aid system. Due to their limited coverage range (less than 2 m) and poor angular sensing resolution, they do not obtain consistent information about the location and the velocity of vehicles on the road [61]. Moreover, ultrasonic sensors suffer disruptions in noisy environments such as roads, streets, and highways. When trying to enlarge the coverage range, the ping/pulse from the emitter can be produced loudly, which is harmful to citizens and the environment. Consequently, sonar systems should remain in their working range, such as parking lots, to accurately detect obstacles [62]. The sonar sensors on the front and rear bumpers should be maintained to be clear of snow, ice, and dirt for maximum accuracy.

## 5. Conclusions and Future Work

Despite the ongoing advancements in automobile technology, one major challenge that has to be overcome is driving safely in severe weather. Driving under less-than-ideal circumstances reduces on-road visibility and impairs the functioning of AV sensors, rendering them vulnerable to possible accidents.

Based on the findings, RADAR sensors seem to align with today’s technology since they can operate beyond adverse weather conditions. The main drawback is that they still need the assistance of a perception system in order to improve decision robustness. LiDAR can be offset to perform in bad weather and, thus, it is still viable. Similarly to RADAR, it requires a perception companion. Cameras can lose their ability to decipher what it observes when lighting changes, weather is severe, or visibility is low. Lightning is a major problem and even obstructions such as dirt-caked signs and cameras will not always be reliable.

On the other hand, LiDAR sensors appear to be more efficient for spacecraft missions, including location and docking, but not for autonomous vehicles. If there are intentions for acquiring active photon generators for automotive purposes, visible wavelength sensors will be useless, since the photon generator covers down to the optical wavelength, while low-cost radars can easily penetrate at incursion situations such as rain, fog, or snow. Numerical studies of a LiDAR sensor simulation tool show that new sensor models can simulate raw data provided by a laser scanner through the pulse laser propagation and energy losses under the weather condition. These attributions will contribute in reducing the cost and time to develop LiDAR applications and to increase performance of such systems. Self-driving vehicles can rely heavily on cameras to perceive the surrounding environment. Therefore, cameras are mostly used due to their low cost, high resolution, and their capability to differentiate between colors. Connecting two visible (VIS) cameras with a predetermined focal distance allows computer stereo vision to be performed; hence, a 3D representation of the scene around the vehicle is produced. However, even in a stereoscopic vision camera system, the estimated depth accuracy is lower than the one obtained from active range finders such as radars and lidars.

Adverse weather conditions can impose challenges and create potential failures to AV sensors. However, with the assistance of satellite for monitoring, the existing infrastructure (e.g., traffic lights) and control centers via mobile devices will be essential for collision avoidance. As a result, prominent semiconductor firms are developing chips that will transform autonomous vehicles into mobile data centers, allowing driverless cars to make crucial decisions in real time.

The paper’s scope is an effort to summarize the weather effects on AV sensors, specifically on RADAR, LiDAR, and camera systems, but the discussion could be expanded. In addition to strategies for minimizing attenuation, the environmental impacts on AV technology, geography, sensor network topology, natural catastrophes, and interferences may be accounted for. What would be even more intriguing is the topic expansion of AV technology effects relative to the environment. Studies on these more expansive dialogues would then contribute to overall sensor information in AV. Although outdoor test environments are the most relevant schemes with high levels of sensor attenuation due to their unpredictable natures, there are additional applications. Scenarios with topology variation, natural disasters, emergencies, physical/internal interference, or lack of human aspects can also contribute to sensor attenuation.

## Figures and Tables

**Table 1:** Summary of autonomous vehicles’ sensors.

Feature	LiDAR	RADAR	Camera	Ultrasonic
Primary Technology	Laser beam	Radio wave	Light	Sound wave
Range	~200 m	~250 m	~200 m	~5 m
Resolution	Good	Average	Very good	Poor
Affected by weather conditions	Yes	Yes	Yes	Yes
Affected by lighting conditions	No	No	Yes	No
Detects speed	Good	Very good	Poor	Poor
Detects distance	Good	Very good	Poor	Good
Interference susceptibility	Good	Poor	Very Good	Good
Size	Bulky	Small	Small	Small

**Table 2:** A list of extinction coefficients for 905 nm and 1550 nm wavelengths subjected to varying levels of humidity.

Extinction Coefficients (904 nm|1550 nm)			
**Percentage (%)**	**1 km Visibility**	**5 km Visibility**	**10 km Visibility**
50	2.333|1.146	0.463|0.227	0.229|0.112
70	2.327|1.137	0.462|0.225	0.228|0.111
100	2.615|1.363	0.519|0.270	0.256|0.133

**Table 3:** Effects of direct and indirect lightning on AV sensors.

AV Sensor	Role(s)	Direct Lightning Effect Levels	Indirect Lightning Effect Levels
RADAR	Electromagnetic	High	Medium
LiDAR	Electrical	High	High
Ultrasonic Sensors	Electromagnetic	High	Medium
GNSS	Electrical, electromagnetic	High	Medium
Camera	Electrical	High	High

**Table 4:** General sound levels.

Sound	Level β (dB)
Stream flow, rustling leaves	15
Watch ticking, soft whisper	20–30
Quiet street noises	40
Normal conversation	45–60
Normal city or freeway traffic	70
Vacuum cleaner	75
Hair dryer	80
Motorcycle, electric shaver	85
Lawn mower, heavy equipment	90
Garbage truck	100
Screaming baby	115
Racing car, loud thunder, rock band	120–130
Jet airplane takeoff from 120 feet	120
Pain threshold	130
Rocket launch from 150 feet	180

**Table 5:** Earth’s atmosphere layers.

Atmospheric Layer	Altitude (km)	Space Weather Effect Level	Applicable AV Sensors
Troposphere	0–14.5	Low–Medium	RADAR, LiDAR, ultrasonic sensors, GNSS, and camera
Stratosphere	14.5–50	Medium	GNSS
Mesosphere	50–85	Medium–High	GNSS
Thermosphere	85–600	High	GNSS
Ionosphere	48–965	High	GNSS
Exosphere	600–10,000	High	GNSS

## Data Availability

Not applicable.

## References

[1] SAE International. https://www.sae.org.

[2] Bimbraw K. Autonomous cars: Past, present and future a review of the developments in the last century, the present scenario and the expected future of autonomous vehicle technology. Proceedings of the 2015 12th International Conference on Informatics in Control, Automation and Robotics (ICINCO).

[3] Daraei M. (2018). Tightly-Coupled LiDAR and Camera for Autonomous Vehicle. Ph.D. Thesis.

[4] History of Autonomous Cars. https://en.wikipedia.org/wiki/History_of_self-driving_cars.

[5] Jernigan M., Alsweiss S., Cathcart J., Razdan R. Conceptual Sensors Testing Framework for Autonomous Vehicles. Proceedings of the 2018 IEEE Vehicular Networking Conference (VNC).

[6] Autonomous Vehicles Cannot Be Test-Driven Enough Miles to Demonstrate Their Safety; Alternative Testing Methods Needed. https://www.rand.org/news/press/2016/04/12.htm.

[7] Kalra N., Paddock S.M. (2016). Driving to safety: How many miles of driving would it take to demonstrate autonomous vehicle reliability?. Transp. Res. Part A Policy Pract..

[8] Rosique F., Navarro P.J., Fernández C., Padilla A. (2019). A Systematic Review of Perception System and Simulators for Autonomous Vehicles Research. Sensors.

[9] Buller W., Wilson B., Garbarino J., Kelly J., Subotic N., Thelen B., Belzowski B. (2018). Radar Congestion Study.

[10] Steinbaeck J., Steger C., Holweg G., Druml N. Next generation radar sensors in automotive sensor fusion systems. Proceedings of the 2017 Sensor Data Fusion: Trends, Solutions, Applications (SDF).

[11] Toker O., Kuhn B. A Python Based Testbed for Real-Time Testing and Visualization using Tis 77 GHz Automotive Radars. Proceedings of the 2019 IEEE Vehicular Networking Conference (VNC).

[12] Vargas J., Alsweiss S., Jernigan M., Amin A., Brinkmann M., Santos J., Razdan R. Development of Sensors Testbed for Autonomous Vehicles. Proceedings of the 2019 IEEE SoutheastCon.

[13] Wojtanowski J., Zygmunt M., Kaszczuk M., Mierczyk Z., Muzal M. (2014). Comparison of 905 nm and 1550 nm semiconductor laser rangefinders’ performance deterioration due to adverse environmental conditions. Opto Electron. Rev..

[14] International Standard IEC 60825-1 (2007). Safety of Laser Products—Part 1: Equipment Classification and Requirements.

[15] Choosing an Ultrasonic Sensor for Proximity or Distance Measurement—Part 1: Acoustic Considerations.FierceElectronics. https://www.fierceelectronics.com/components/choosing-ultrasonic-sensor-for-proximity-or-distance-measurement-part-1-acoustic-0.

[16] Nagaoka S., Raihanul I., Okajima K., Ito R., Watanabe K., Ito T. (2018). Evaluation of Attenuation of Ultrasonic Wave in Air to Measure Concrete Roughness Using Aerial Ultrasonic Sensor. Int. J. GEOMATE.

[17] GPS: The Global Positioning System. https://www.gps.gov/systems/gps.

[18] Chang W., Chen C., Hung Y. (2019). Tracking by parts:A bayesian approach with component collaboration. IEEE Trans. Syst. Man Cybern. Part B Cybern..

[19] Kalal Z., Mikolajczyk K., Matas J. (2012). Tracking-learning-detection. IEEE Trans. Pattern Anal. Mach. Intell..

[20] Pepik B., Gehler P., Stark M., Schiele B. (2012). 3D^2^PM–3D Deformable part models. Computer Vision—ECCV.

[21] Prisacariu V.A., Reid I.D. (2012). PWP3D: Real-time segmentation and tracking of 3d objects. Int. J. Comput. Vis..

[22] Vatavu A., Danescu R., Nedevschi S. (2015). Stereovision-based multiple object tracking in traffic scenarios using free-form obstacle delimiters and particle filters. IEEE Trans. Intell. Transp. Syst..

[23] Erbs F., Barth A., Franke U. Moving vehicle detection by optimal segmentation of the dynamic stixel world. Proceedings of the 2011 IEEE Intelligent Vehicles Symposium (IV).

[24] Olmeda D., de la Escalera A., Armingol J.M. (2011). Far infrared pedestrian detection and tracking for night driving. Robotica.

[25] Gade R., Moeslund T.B. (2014). Thermal cameras and applications: A survey. Mach. Vis. Appl..

[26] González A., Fang Z., Socarras Y., Serrat J., Vázquez D., Xu J., López A., González A., Fang Z., Socarras Y. (2016). Pedestrian Detection at Day/Night Time with Visible and FIR Cameras: A Comparison. Sensors.

[27] Sun H., Wang C., Wang B. Night Vision Pedestrian Detection Using a Forward-Looking Infrared Camera. Proceedings of the 2011 IEEE International Workshop on Multi-Platform/Multi-Sensor Remote Sensing and Mapping.

[28] John V., Mita S., Liu Z., Qi B. Pedestrian detection in thermal images using adaptive fuzzy C-means clustering and convolutional neural networks. Proceedings of the 2015 IEEE 14th IAPR International Conference on Machine Vision Applications (MVA).

[29] Forslund D., Bjarkefur J. Night vision animal detection. Proceedings of the 2014 IEEE Intelligent Vehicles Symposium Proceedings.

[30] Hadj-Bachir M., Souza P. (2019). LIDAR Sensor Simulation in Adverse Weather Condition for Driving Assistance Development (hal-01998668f), Hal.archives-ouvertes.fr. https://hal.archives-ouvertes.fr/hal-01998668.

[31] Bertoldo S., Lucianaz C., Allegretti M. 77 GHz automotive anti-collision radar used for meteorological purposes. Proceedings of the 2017 IEEE-APS Topical Conference on Antennas and Propagation in Wireless Communications (APWC).

[32] (2005). ITU, Specific Attenuation Model for Rain for Use in Prediction Methods. https://www.itu.int/rec/R-REC-P.838.

[33] Gourova R., Krasnov O., Yarovoy A. Analysis of rain clutter detections in commercial 77 GHz automotive radar. Proceedings of the 2017 European Radar Conference (EURAD).

[34] Hassen A.A. (2007). Indicators for the Signal Degradation and Optimization of Automotive Radar Sensors under Adverse Weather Conditions. Ph.D. Thesis.

[35] Huang J., Jiang S., Lu X. (2001). Rain backscattering properties and effects on the radar performance at mm wave band. Int. J. Infrared Millim. Waves.

[36] Kaplan E., Hegarty C. (2005). Understanding GPS: Principles and Applications.

[37] Zang S., Ding M., Smith D., Tyler P., Rakotoarivelo T., Kaafar M.A. (2019). The Impact of Adverse Weather Conditions on Autonomous Vehicles: How Rain, Snow, Fog, and Hail Affect the Performance of a Self-Driving Car. IEEE Veh. Technol. Mag..

[38] Fu X., Huang J., Ding X., Liao Y., Paisley J. (2017). Clearing the skies: A deep network architecture for single-image rain removal. IEEE Trans. Image Process..

[39] Gultepe I. (2008). Fog and Boundary Layer Clouds: Fog Visibility and Forecasting.

[40] Awan M.S., Leitgeb E., Loeschnig M., Nadeem F., Capsoni C. Spatial and Time Variability of Fog Attenuations for Optical Wireless Links in the Troposphere. Proceedings of the 2009 IEEE 70th Vehicular Technology Conference Fall.

[41] What is Humidity? How is Humidity Measured?. https://www.edinformatics.com/math_science/what-is-humidity.html.

[42] Vehicles and Lightning—National Lightning Safety Institute. http://lightningsafety.com/nlsi_pls/vehicle_strike.html.

[43] Gaynutdinov R.R., Chermoshentsev S.F. Electromagnetic stability of an unmanned aerial vehicle at the indirect effect of a lightning discharge. Proceedings of the 2017 International Multi-Conference on Engineering, Computer and Information Sciences (SIBIRCON).

[44] The Thunder Mechanism—National Lightning Safety Institute. http://lightningsafety.com/nlsi_info/thunder.html.

[45] Why is Sound Attenuation Greater in Dry Air than Humid Air?. https://physics.stackexchange.com/questions/507729/why-is-sound-attenuation-greater-in-dry-air-than-humid-air.

[46] Zeng C., Richardson M., King D. (2017). The impacts of environmental variables on water reflectance measured using a lightweight unmanned aerial vehicle (UAV)-based spectrometer system. ISPRS J. Photogramm. Remote. Sens..

[47] Blowing K. Dust and Dust Storms: One of Arizona’s Most Underrated Weather Hazards. http://www.atmo.arizona.edu/images/news/Aish_Article.pdf.

[48] World Meteorological Organization (2019). Sand and Dust Storms. https://public.wmo.int/en/our-mandate/focus-areas/environment/SDS.

[49] Starr W.J., Lattimer B.Y. (2013). Evaluation of Navigation Sensors in Fire Smoke Environments. Fire Technol..

[50] Earth’s Atmospheric Layers, NASA. https://www.nasa.gov/mission_pages/sunearth/science/atmosphere-layers2.html.

[51] Sreeja V. (2016). Impact and mitigation of space weather effects on GNSS receiver performance. Geosci. Lett..

[52] Heinzler R., Schindler P., Seekircher J., Ritter W., Stork W. Weather Influence and Classification with Automotive Lidar Sensors. Proceedings of the 2019 IEEE Intelligent Vehicles Symposium (IV).

[53] Lidar vs. Radar: Pros and Cons for Autonomous Driving. https://archer-soft.com/blog/lidar-vs-radar-pros-and-cons-autonomous-driving.

[54] Kulemin G.P. (1999). Influence of propagation effects on millimeter wave radar operation. Radar Sens. Technol..

[55] Wallace H.B. (1988). Millimeter-wave propagation measurements at the ballistic research laboratory. IEEE Trans. Geosci. Remote Sens..

[56] Battan L.J. (1971). Radar attenuation by wet ice spheres. J. Appl. Meteorol..

[57] Lhermitte R. (1990). Attenuation and scattering of millimeter wavelength radiation by clouds and precipitation. J. Atmos. Ocean. Technol..

[58] Pozhidaev V.N. (2010). Estimation of attenuation and backscattering of millimeter radio waves in meteorological formations. J. Commun. Technol. Electron..

[59] Yu X., Marinov M. (2020). A Study on Recent Developments and Issues with Obstacle Detection Systems for Automated Vehicles. Sustainability.

[60] Hane C., Sattler T., Pollefeys M. Obstacle detection for self-driving cars using only monocular cameras and wheel odometry. Proceedings of the International Conference on Intelligent Robots and Systems.

[61] Rasshofer R.H., Gresser K. (2005). Automotive radar and lidar systems for next generation driver assistance functions. Adv. Radio Sci..

[62] Langer D., Thorpe C.E. Sonar Based Outdoor Vehicle Navigation and Collsion Avoidance. Proceedings of the IEEE/RSJ International Conference on Intelligent Robots and Systems.

